# A Prediction Model for Sustained Casing Pressure in B-Annulus for Shale Gas Well Induced by the Leakage of the Micro-Annulus at the Casing-Cement Sheath Interface

**DOI:** 10.1021/acsomega.6c07100

**Published:** 2026-09-21

**Authors:** Honglin Xu, Jie Wen, Nian Peng, Ce Zhang, Jiawei Lu, Shilin Xiang, Zhi Zhang

**Affiliations:** † School of Petroleum and Natural Gas Engineering, 66564 Chongqing University of Science and Technology, Shapingba, Chongqing 401331, China; ‡ State Key Laboratory of Oil and Gas Reservoir Geology and Exploitation, Southwest Petroleum University, Chengdu 610500, China

## Abstract

This paper proposes a prediction method for sustained casing pressure (SCP) in the B-annulus induced by gas leakage at the casing-cement sheath interface (CCSI). The cross-sectional area of microscopic leakage channels at the CCSI is first quantitatively characterized based on micro-contact finite-element simulation. Then, based on parallel-plate theory and the laminar-flow theory of incompressible viscous fluids as an engineering approximation, a gas leakage-rate prediction model through the CCSI is established. Finally, considering both the variation in gas leakage rate at the CCSI and gas accumulation at the wellhead, a coupled prediction model for gas leakage rate and SCP is developed using a recurrence method. The case study shows that the relative errors for the predicted stabilized SCP and SCP stabilization time are 7.09% and 1.9%, respectively, which validates the proposed model. Furthermore, a systematic parameter sensitivity analysis is conducted on the evolution laws of SCP in the B-annulus of a shale gas well. The results show that the elastic modulus and Poisson’s ratio of the cement sheath exert insignificant effects on SCP evolution. The roughness and axial leakage length at the CCSI significantly affect the SCP stabilization time by altering gas leakage resistance. Variations in the internal pressure of the production casing and wellbore temperature exert moderate effects on SCP evolution mainly by changing the interfacial contact pressure. The density of the annular protection fluid and the formation pressure, by altering the driving pressure difference for interfacial gas leakage, not only significantly affect the stabilization time of SCP but also evidently influence the final stabilized SCP value. To mitigate SCP risk in the B-annulus, reducing the roughness and ensuring sufficient effective cementing length at the CCSI, adopting cement sheaths with low elastic modulus and high Poisson’s ratio, and appropriately increasing the density of the annular protection fluid are recommended.

## Graphical abstract

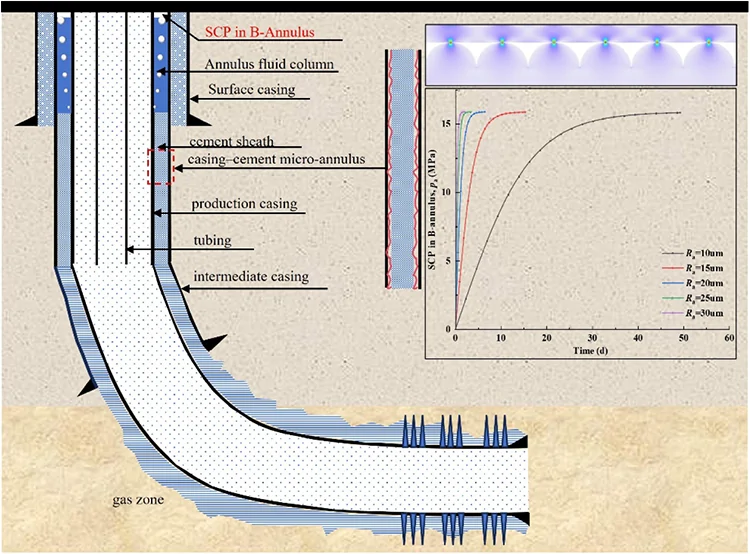

## 1 Introduction

Shale gas reservoirs are universally characterized by extremely low porosity (4–6%) and permeability (<0.001 × 10^–3^ μm^2^). Horizontal well drilling combined with volumetric fracturing constitutes the core technology for the efficient development of shale gas.^1^ The cement sheath serves as a critical physical barrier ensuring wellbore seal integrity in shale gas wells. However, high-rate and high-pressure volumetric fracturing operations tend to induce microcracks in the cement sheath or interfacial micro-annuli. Among these, the micro-annulus at the casing-cement sheath interface (CCSI) is identified as the dominant leakage pathway for cement sheath sealing failure in shale gas wells.^2^

As illustrated in Figure 1, once a micro-annulus forms at the CCSI, shallow gas or reservoir gas can easily migrate through this leakage pathway into the annulus between the intermediate casing and production casing (B-annulus), accumulate, and consequently induce SCP. Statistics indicate that 40.7% of the producing wells in a demonstration zone located on the southeastern margin of the Sichuan Basin present SCP in B-annulus exceeding 10 MPa, while the percentage of wells exhibiting SCP rose from 19.04% to 44.64% before and after fracturing operations.^3^ Among the 63 deep shale gas wells statistically analyzed in the western Chongqing block, the proportion of wells with SCP in the B-annulus amounts to 39.7%. SCP in shale gas wells not only impairs gas production and operational safety but may also lead to well abandonment and even public safety hazards in severe cases.^4^ Therefore, quantitative characterization of the microscopic leakage channel dimensions at the cement sheath interface, as well as the establishment of a prediction model for SCP induced by interfacial microscale gas leakage, is of considerable theoretical significance and practical engineering value for revealing the SCP evolution mechanism, identifying the root causes of SCP, and controlling and mitigating the associated hazards in shale gas wells.

**Figure 1:**
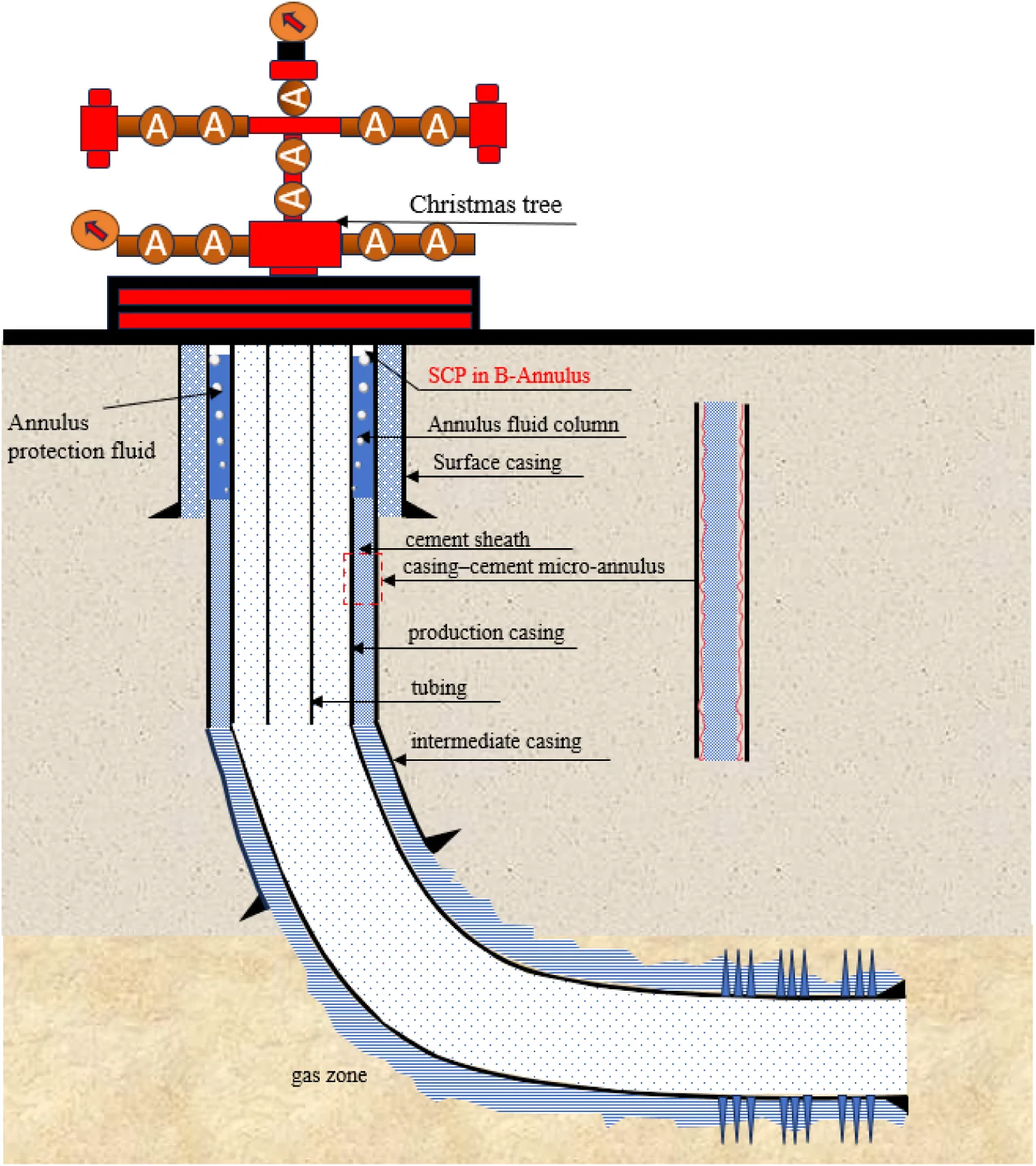
Schematic diagram of SCP in B-annulus induced by micro-annulus leakage at the cement sheath interface of a shale gas well.

At present, research on microannuli at the CCSI mainly focuses on two aspects: the formation mechanism of microannuli and the quantitative characterization of microannulus geometry. Based on experimental investigations, the formation of microannuli at the CCSI was observed following cyclic loading of casing pressure from 1000 to 8000 psi and subsequent reduction to 1000 psi.^5^ It was identified that the thermal expansion of the wellbore during heating and the thermal contraction during cooling can result in interfacial debonding at the CCSI.^6^ It was demonstrated that microannuli can be induced in the cement sheath under high confining pressure when the internal casing pressure is subjected to variation.^7^ Cheng et al. further proposed an evaluation approach for the interfacial compatibility of the cement sheath based on the Dundurs composite parameter method.^8^ Although experimental methods can directly observe the initiation of microannuli in the cement sheath under the action of various mechanical factors, they can hardly quantitatively characterize the geometrical dimensions of interfacial microannuli from a mechanical mechanism perspective. Moreover, experimental methods feature long testing cycles and high costs; therefore, they are generally employed only as a verification approach.

Mechanical analysis of the casing-cement sheath-formation composite system constitutes the primary method for investigating mechanical damage evolution in the cement sheath. Interfacial debonding can be qualitatively determined via stress calculation of the cement sheath combined with strength criteria, while the magnitude of micro-annuli can be quantitatively obtained by calculating the incompatible deformation at the CCSI. In the realm of theoretical modeling, Bois developed an early mechanical model for the cement sheath based on the multilayer thick-walled cylinder theory. It was concluded that interfacial debonding occurs when the radial tensile stress induced in the cement sheath at the interface by plastic deformation exceeds the interfacial bonding strength.^9^ Niu et al. developed a thermo-poroelastic mechanical model for evaluating cement sheath integrity. They analyzed the evolution law of effective stress in the cement sheath induced by thermal loads resulting from the temperature difference between the injected fluid inside the casing and the formation as well as the thermal percolation effect and put forward the failure criterion of the cement sheath.^10^ Yin et al. proposed a novel thermo-mechanical coupled model for the wellbore system. This model accounts for the interfacial bonding behavior and thermal effects at the cemented interfaces, enabling the prediction of temperature, stress displacement fields, and interfacial debonding responses during injection or fracturing operations in high-temperature reservoirs.^11^ Wang et al. established a novel mechanical model for the casing-cement interface and cement-formation interface in shale gas wells based on fracture mechanics and discussed the influences of casing and cement sheath parameters on interfacial stresses and fracture aperture.^12^ Su et al. developed a theoretical model for evaluating the sealing integrity of the cement-formation interface combined with a self-developed sealing evaluation apparatus for the cement sheath interface and proposed the mechanical performance requirements for Young’s modulus (*E*_c_) and Poisson’s ratio (*V*_c_) to ensure the sealing integrity of the cement sheath.^13^ Chu et al. calculated the micro-annulus size in the casing-cement-formation system under varying casing internal pressure, yet temperature variations were not considered in their analysis.^14^ However, owing to the coupled effects of interfacial contact nonlinearity, material nonlinearity, and boundary load nonlinearity in the casing-cement sheath-formation composite system, it is difficult for purely theoretical models to reliably and quantitatively characterize the micro-annulus dimension at the cement sheath interface after fracturing. Given that numerical simulation enables a more convenient and efficient solution to the complex nonlinearities involving material constitutive models, loading conditions, and boundaries in the casing-cement sheath-formation composite system, researchers have carried out studies on cement sheath micro-annuli using numerical simulation methods. Zhu et al. utilized cohesive elements to simulate the interfacial debonding failure of the cemented interface during hydraulic fracturing.^15^ Gu et al. developed a finite element model for evaluating the interfacial sealing performance of the cement sheath and investigated the influences of well trajectory, casing eccentricity, and cement sheath porosity on the failure behavior at the cement sheath interface.^16^ Li et al. established a numerical model of the casing-cement sheath system based on the cyclic dynamics method, overcoming the singularity problem at the interface encountered in conventional numerical methods based on the continuity assumption.^17^ Ramadan et al. established a numerical model for gas flow within cement sheaths containing micro-annuli and investigated the effects of micro-annulus size, cement matrix permeability, cement column length, and cement porosity on gas leakage. However, no computational basis was provided for determining the micro-annulus scale parameters.^18^ Xi et al. established a numerical model of the cement sheath considering transient thermo-mechanical coupling effects and a numerical model for cumulative plastic deformation of the cement sheath under cyclic loading for shale gas wells. It was pointed out that the early stage of fracturing in shale gas wells represents the most critical dangerous stage for the cement sheath, and the use of flexible cement, as well as increasing cohesion and internal friction angle, is beneficial to protecting the cement sheath.^19^ Fan et al. established a finite element model for interfacial crack propagation in the cement sheath using the extended finite element method and indicated that crack propagation in the cement sheath is governed by interfacial bonding conditions, cement sheath formation quality, and material properties. They concluded that a higher interfacial bonding strength helps reduce the propagation length of interfacial cracks.^20^ However, whether in theoretical models of the casing-cement sheath-formation system or numerical models of the cement sheath, almost all existing studies treat the cement sheath interface as perfectly smooth. In practice, after micro-annuli form at the casing-cement interface due to debonding, the interface is in an elastic-plastic microscopic contact state under the effects of wellbore internal pressure, temperature variations, and in-situ stress. Therefore, it is necessary to establish a microscopic contact model at the casing-cement interface to quantitatively characterize the micro-annulus size.

At present, the mechanism models for SCP in gas wells mainly fall into three categories: thermal expansion-induced SCP models, tubing leakage-induced SCP models, and cement sheath leakage-induced SCP models. Thermal expansion-induced SCP mainly occurs during the initial stage of well completion and production and can generally be relieved by pressure bleeding, which is fundamentally different in mechanism from SCP induced by wellbore leakage.^21,22^ Regarding research on SCP induced by wellbore leakage, Wang et al. analyzed the flow, leakage, and accumulation of gas in the wellbore and proposed a prediction model for SCP induced by tubing string leakage that accounts for real gas effects.^23^ Luo et al. established a prediction model for SCP caused by completion string leakage based on the throttling model, gas-liquid two-phase flow model, and gas equation of state.^24^ Luo et al. developed a prediction model for SCP induced by cement sheath seepage in shale gas wells using porous media seepage mechanics and the gas equation of state for accumulated gas in the wellhead annulus.^25^ Lima et al. investigated the effect of relative permeability on the hydraulic aperture of potential leakage channels, providing a basis for analyzing gas migration and SCP in the cement sheath.^26^ Based on the above studies, it is found that SCP caused by cement sheath damage usually occurs in the B-annulus, while that induced by tubing leakage typically appears in the A-annulus. The difference in physical mechanisms between the two types of leakage-induced SCP is mainly reflected in the distinct gas leakage modes in the tubing string and cement sheath, whereas the gas accumulation behavior at the wellhead is similar. However, since the micro-annulus at the cementing sheath of shale gas wells is the main channel for cement sheath leakage, existing models for annular pressure buildup induced by cement sheath leakage only consider the equivalent seepage effect of the cement sheath body and are therefore not applicable to the prediction of SCP in the B-annulus of shale gas wells.

This paper proposes a prediction method for SCP induced by gas leakage at the casing-cement interface based on microscopic contact simulation. Based on the elastic-plastic normal microscopic contact simulation at the casing-cement interface, this method obtains the quantitative relationship between the macroscopic interfacial contact pressure and the actual elastic-plastic contact area of asperities, thereby quantitatively characterizing the cross-sectional area of the microscopic leakage channels at the casing-cement interface. Based on the parallel plate theory in fluid mechanics and the laminar flow theory of incompressible viscous fluids, a prediction model for the microscopic gas leakage rate at the casing-cement interface of gas wells is established. Considering both the variation of gas leakage rate at the casing-cement interface and the gas accumulation effect at the wellhead, a coupled prediction model of interfacial leakage rate and SCP is established using the recursive method. The reliability of the model was verified using field-measured B-annulus pressure data, and a systematic parametric sensitivity analysis was conducted. The effects of cement sheath material parameters, interfacial parameters, and wellbore engineering parameters on the evolution of SCP in the B-annulus of shale gas wells were systematically investigated, and corresponding prevention and control measures were proposed, providing important technical support for the safe and efficient development of shale gas wells.

## 2 Model Establishment and Solution

### 2.1 Calculation Model of Contact Pressure at the Casing-Cement Interface

After cementing in shale gas wells, the production casing, cement sheath, and formation rock mass can be regarded as a composite cylinder, as shown in Figure 2. During production of gas wells, variations in the inner pressure of the production casing, external formation pressure, and temperature of the composite cylinder will generate contact pressure at both the casing-cement interface and the cement-formation interface. Among them, the contact pressure at the casing-cement interface directly affects the actual leakage channel size of the interfacial microannulus. Xu et al. established a mechanical model of the casing-cement sheath-formation assembly under plane strain conditions based on the multi-layer thick-walled cylinder theory. The contact pressures at the two interfaces of the cement sheath can be calculated according to the continuity condition of radial displacement at the interfaces.^27^

**Figure 2:**
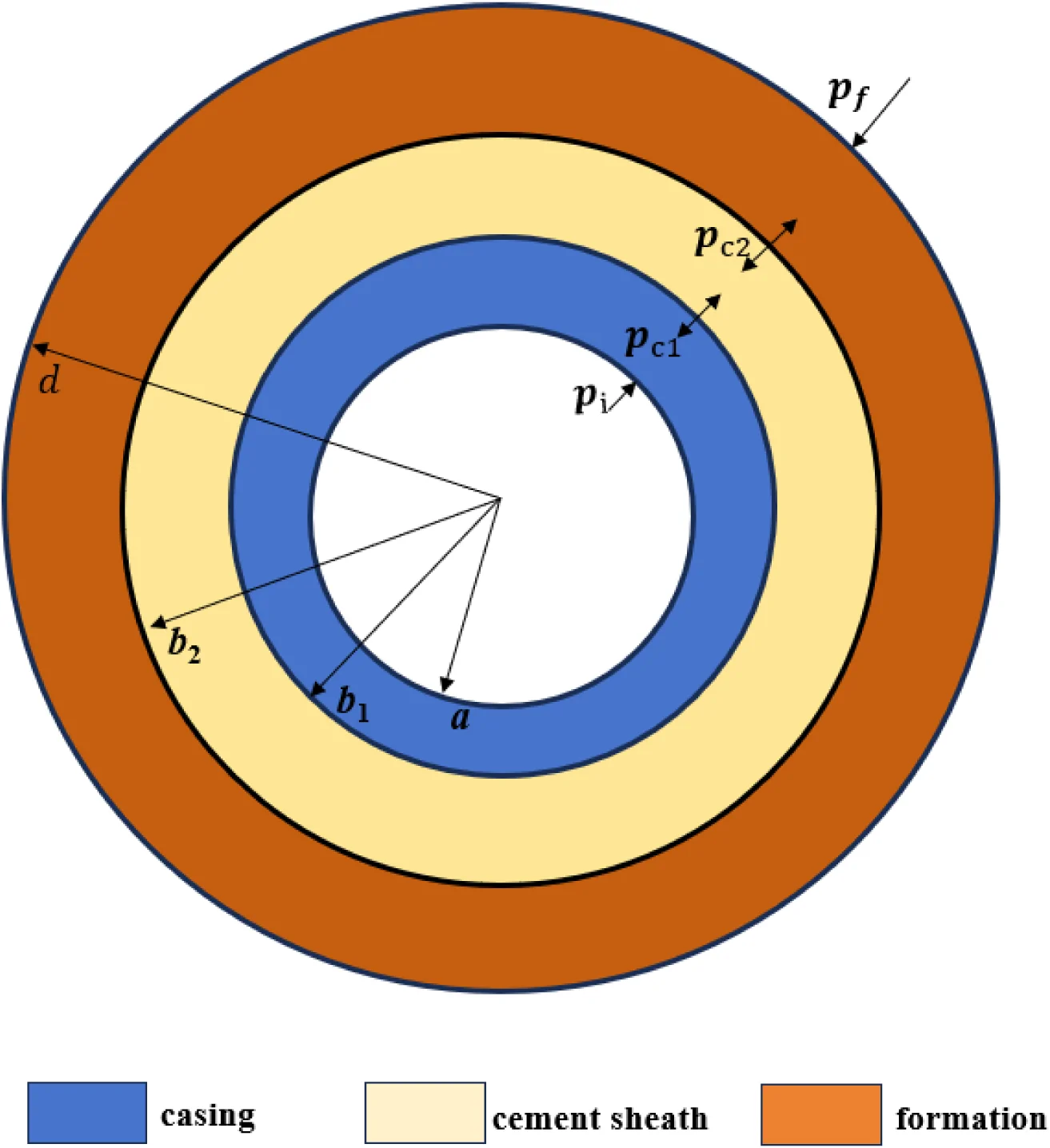
Mechanical model of casing-cement sheath-formation composite system.

Considering the combined effects of casing internal pressure variation *p*_i_, casing-cement interface contact pressure *p*_c1_, and wellbore temperature variation Δ*T*, the radial deformation of the casing outer wall is given by:

1 $$\delta_{\mathrm{rso}}=\frac{b_{1}}{E_{s}}\left[\left(1-\upsilon_{s}^{2}\right)\left(\frac{2a^{2}}{{b_{1}}^{2}-a^{2}}p_{i}-\frac{{b_{1}}^{2}+a^{2}}{{b_{1}}^{2}-a^{2}}p_{c1}\right)+\left(\upsilon_{s}+\upsilon_{s}^{2}\right)p_{c1}+\left(1+\upsilon_{s}\right)\alpha_{s}E_{s}\Delta T\right]$$

Considering the combined effects of *p*_c1_, cement-formation interface contact pressure *p*_c2_, and Δ*T*, the radial deformations of the inner and outer walls of the cement sheath are given by:

2 $$\delta_{\mathrm{rci}}=\frac{b_{1}}{E_{c}}\left[\left(1-\upsilon_{c}^{2}\right)\left(\frac{{b_{2}}^{2}+{b_{1}}^{2}}{{b_{2}}^{2}-{b_{1}}^{2}}p_{c1}-\frac{2{b_{2}}^{2}}{{b_{2}}^{2}-{b_{1}}^{2}}p_{c2}\right)+\left(\upsilon_{c}+\upsilon_{c}^{2}\right)p_{\mathrm{cl}}+\left(1+\upsilon_{c}\right)\alpha_{c}E_{c}\Delta T\right]$$

3 $$\begin{array}{lll} \delta_{\mathrm{rco}} & = & \frac{b_{2}}{E_{c}}\left\{\left(1-\upsilon_{c}^{2}\right)\left[p_{c1}\left(\frac{2{b_{1}}^{2}}{{b_{2}}^{2}-{b_{1}}^{2}}\right)-p_{c2}\left(\frac{{b_{2}}^{2}+{b_{1}}^{2}}{{b_{2}}^{2}-{b_{1}}^{2}}\right)\right]+p_{c2}\left(\upsilon_{c}+\upsilon_{c}^{2}\right)\right\}\\ & & +\left[\left(1+\upsilon_{c}\right)b_{2}\alpha_{c}\Delta T\right] \end{array}$$

Considering the combined effects of *p*_c2_, formation pressure variation *p*_f_, and Δ*T*, the radial deformation of the inner wall of the formation is given by:

4 $$\begin{array}{lll} \delta_{\mathrm{rfi}} & = & \frac{b_{2}}{E_{f}}\left\{\left(1-\upsilon_{f}^{2}\right)\left[p_{c2}\left(\frac{{b_{2}}^{2}+d^{2}}{d^{2}-{b_{2}}^{2}}\right)-p_{f}\left(\frac{2d^{2}}{d^{2}-{b_{2}}^{2}}\right)\right]+p_{c2}\left(\upsilon_{f}+\upsilon_{f}^{2}\right)\right\}\\ & & +\left[\left(1+\upsilon_{f}\right)b_{2}\alpha_{f}\Delta T\right] \end{array}$$

According to the continuity condition of radial displacement at the casing-cement interface, the two radial deformations in eqs 1 and 2 should be equal, yielding:

5 $$\begin{array}{l} \left[\begin{array}{l} E_{c}\left(\upsilon_{s}+\upsilon_{s}^{2}\right)-\frac{{b_{1}}^{2}+a^{2}}{{b_{1}}^{2}-a^{2}}E_{c}\left(1-\upsilon_{s}^{2}\right)\\ -\frac{{b_{2}}^{2}+{b_{1}}^{2}}{{b_{2}}^{2}-{b_{1}}^{2}}E_{s}\left(1-\upsilon_{c}^{2}\right)-E_{s}\left(\upsilon_{c}+\upsilon_{c}^{2}\right) \end{array}\right]p_{c1}+\frac{2{b_{2}}^{2}}{{b_{2}}^{2}-{b_{1}}^{2}}E_{s}\left(1-\upsilon_{c}^{2}\right)p_{c2}\\ =\alpha_{c}E_{c}E_{s}\Delta T\left(1+\upsilon_{c}\right)-\alpha_{s}E_{s}E_{c}\Delta T\left(1+\upsilon_{s}\right)-\frac{2a^{2}}{{b_{1}}^{2}-a^{2}}E_{c}\left(1-\upsilon_{s}^{2}\right)p_{i} \end{array}$$

Similarly, according to the continuity condition of radial displacement at the cement-formation interface, the two radial deformations in eqs 3 and 4 should also be equal, yielding:

6 $$\begin{array}{l} \frac{2{b_{1}}^{2}}{{b_{2}}^{2}-{b_{1}}^{2}}E_{f}\left(1-\upsilon_{c}^{2}\right)p_{c1}+\left[\begin{array}{l} E_{f}\left(\upsilon_{c}+\upsilon_{c}^{2}\right)-\frac{{b_{2}}^{2}+{b_{1}}^{2}}{{b_{2}}^{2}-{b_{1}}^{2}}E_{f}\left(1-\upsilon_{c}^{2}\right)\\ -\frac{d^{2}+{b_{2}}^{2}}{d^{2}-{b_{2}}^{2}}E_{c}\left(1-\upsilon_{f}^{2}\right)-E_{c}\left(\upsilon_{f}+\upsilon_{f}^{2}\right) \end{array}\right]p_{c2}\\ =\alpha_{f}E_{f}E_{c}\Delta T\left(1+v_{f}\right)-\alpha_{c}E_{c}E_{f}\Delta T\left(1+v_{c}\right)-\frac{2d^{2}}{d^{2}-{b_{2}}^{2}}E_{c}\left(1-v_{f}^{2}\right)p_{f} \end{array}$$

Thus, by simultaneously solving eqs 5 and 6, the casing-cement interface contact pressure *p*_c1_ can be obtained.

In eqs 1–6, a is the inner radius of the casing in mm; *b*_1_ is the outer radius of the casing or the inner radius of the cement sheath in mm; *b*_2_ is the outer radius of the cement sheath or the inner radius of the formation in mm; *d* is the outer radius of the formation in mm; *p*_i_ is the variation of the casing internal pressure in MPa; *p*_c1_ is the contact pressure at the casing-cement sheath interface in MPa; *p*_c2_ is the contact pressure at the cement sheath-formation interface in MPa; *p*_f_ is the variation of the formation pressure in MPa; *E*_s_, *E*_c_, and *E*_f_ are the elastic moduli of the casing, cement sheath, and formation, respectively, in MPa; ν_s_, ν_c_, and ν_f_ are the Poisson’s ratios of the casing, cement sheath, and formation, respectively, which are dimensionless; α_s_, α_c_, and α_f_ are the linear thermal expansion coefficients of the casing, cement sheath, and formation, respectively, in 1/°C; δ_rso_, δ_rci_, δ_rco_, and δ_rfi_ are the radial deformations of the casing outer wall, cement sheath inner wall, cement sheath outer wall, and formation inner wall, respectively, in mm; and Δ*T* is the average variation of the wellbore temperature in °C.

### 2.2 Finite Element Model for Microscopic Contact at the Casing-Cement Interface

The contact at the casing-cement sheath interface is essentially a microscopic contact problem between two rough surfaces. In rough-surface contact simulations, various asperity geometries may be considered, including hemispherical, ellipsoidal, cylindrical, sinusoidal, and conical asperities. Among these, the spherical asperity contact model is the most widely used and has been extensively employed in statistical models, fractal models, and finite-element contact simulations to determine the relationship between the real contact area and the contact load during the elastoplastic contact of rough surfaces.^28^

According to the equivalent simulation principle in contact mechanics, the microscopic contact between two rough surfaces can be equivalent to the contact between a spherical asperity surface with equivalent elastic modulus E* and a rigid plane, where E* can be calculated as follows:^29^

7 $$E^{*}=\frac{E_{s}E_{c}}{E_{s}\left(1-v_{c}^{2}\right)+E_{c}\left(1-v_{s}^{2}\right)}$$

where E* is the equivalent elastic modulus, in MPa.

Figure 3 shows the schematic diagram of simulated microscopic contact between a spherical asperity rough surface and a rigid plane in the xoz cross section. Figure 3(a) represents the initial contact state, at which the casing-cement interface contact pressure *p*_c1_ is zero; Figure 3(b) shows the microscopic contact state of interfacial asperities under the actual casing-cement interface contact pressure *p*_c1_. Obviously, the contact projection of spherical asperities at the casing-cement interface is approximately circular, and the area of this circle is inherently related to the leakage channel size of the sealing surface. Therefore, to obtain the actual contact area of spherical asperities under *p*_c1_, a finite element model for simulating the casing-cement interface microscopic contact is first established.

**Figure 3:**
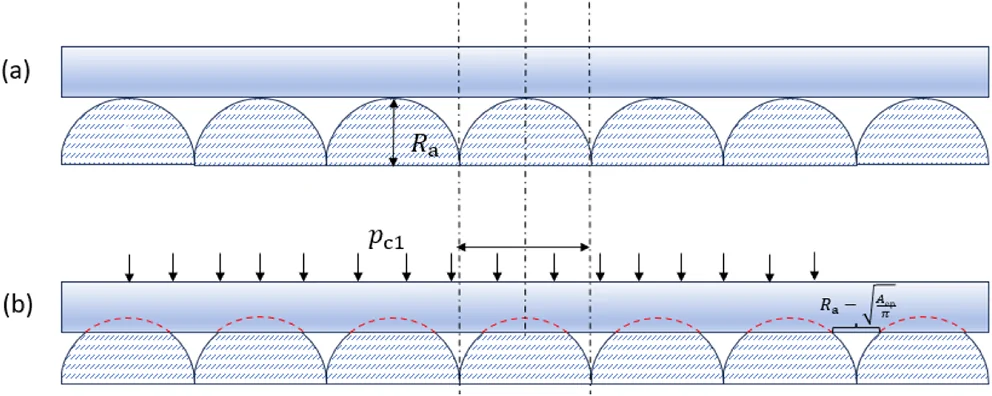
Schematic diagram of microscopic contact between a spherical asperity rough surface and a rigid plane in the xoz cross-section: (a) initial contact state; (b) micro-contact state under the actual interfacial contact pressure *p*_c1_.

Considering the axisymmetry of the spherical asperity, a 2D finite element model for microscopic contact simulation between the spherical asperity and the rigid flat plate (Figure 4) is established to improve computational efficiency. The rigid flat plate is sized at 280 × 35 μm, and the asperity radius is 20 μm. The elastic modulus of the asperity is set to E*, and an elastoplastic constitutive relation is used to simulate its deformation. To balance computational efficiency and accuracy, a variable-density mesh method is adopted for the model, with mesh refinement applied in the contact region. The contact pair between the asperity and the rigid flat plate is defined as gap-free and tangentially non-slip. The contact pressure *p*_c1_ is applied to the upper surface of the rigid flat plate, and fixed constraints are imposed on both sides of the rigid flat plate and the bottom of the asperity. To accelerate the iteration speed, the augmented Lagrangian method is adopted as the iterative algorithm. Thus, the elastoplastic actual contact area of a single spherical asperity at the casing-cement interface under the radial contact pressure *p*_c1_ can be obtained.

8 $$A_{\mathrm{ep}}=f\left(p_{c1}\right)$$

where *A*_ep_ is the elastoplastic actual contact area of a single spherical asperity, μm^2^; and *p*_c1_ is the contact pressure at the CCSI, MPa.

**Figure 4:**
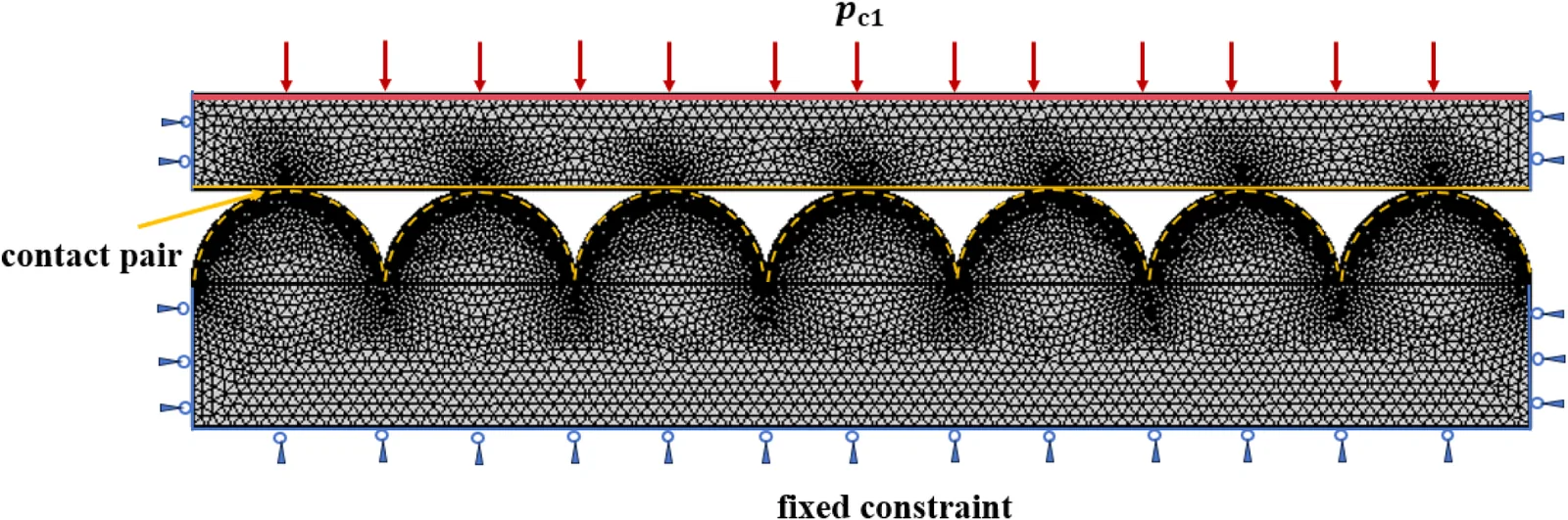
Meshing for microscopic contact simulation at the CCSI.

### 2.3 Leakage Rate Prediction Model for the Casing-Cement Sheath Interface

After obtaining the actual contact area *A*_ep_ of a single spherical asperity, the circumferential width *W* of the microscopic leakage channel at the casing-cement contact interface can be derived from the geometric relationship in Figure 3 as follows:

9 $$W=\frac{2000\pi b_{1}}{R_{a}}\left(R_{a}-\sqrt{\frac{A_{\mathrm{ep}}}{\pi }}\right)$$

where *W* is the circumferential width of the microscopic leakage channel at the casing-cement sheath interface, in μm; and *R*_a_ is the circumferential surface roughness of the cement sheath, in μm.

Meanwhile, the average height *H* of the microscopic leakage channel at the CCSI can be equivalently obtained by calculating the cross-sectional area using the integral method.

10 $$H=\frac{\left(2R_{a}-\sqrt{\frac{A_{\mathrm{ep}}}{\pi }}\right)\sqrt{R_{a}^{2}-\frac{A_{\mathrm{ep}}}{\pi }}+\left(\arcsin\frac{1}{R_{a}}\sqrt{\frac{A_{\mathrm{ep}}}{\pi }}-\frac{\pi }{2}\right)R_{a}^{2}}{2\left(R_{a}-\sqrt{\frac{A_{\mathrm{ep}}}{\pi }}\right)}$$

where *H* is the average height of the microscopic leakage channel at the CCSI, in μm.

According to previous studies on industrial sealing technology and micrometer-scale flow like metal-to-metal sealing, the gas flow through the micro gaps between sealing surfaces can generally be regarded as the laminar flow of an incompressible viscous fluid.^30,31^ Furthermore, based on the fluid mechanics parallel plate theory and the laminar flow theory of incompressible viscous fluids, the volumetric leakage rate of gas through the CCSI can be calculated:^32^

11 $$Q_{v}=\frac{10^{-18}WH^{3}\left(p_{f}-p_{a}-10^{-6}\rho_{l}gH_{p}\right)}{12L_{c}\eta }$$

where *Q*_v_ is the gas leakage volumetric rate at the CCSI, cm^3^/s; *p*_a_ is the SCP in the B-annulus, MPa; ρ_l_ is the density of the annulus protective fluid, kg/m^3^; *H*_p_ is the height of the annulus protective fluid column, m; *L*_c_ is the axial contact length of the casing-cement sheath cementing interface along the well depth direction, m; and η is the dynamic viscosity of the production-zone natural gas, Pa·s.

### 2.4 Prediction Model for SCP Caused by Gas Leakage at Casing-Cement Sheath Interface

Gas leaks through the CCSI, migrates upward through the annulus protective fluid to the wellhead, and accumulates, causing the SCP in the B-annulus to rise continuously. The continuous rise of SCP in B-annulus will gradually reduce the driving pressure difference for gas leakage at the interface, and the leakage rate will decrease accordingly. When the driving pressure difference for leakage gradually approaches zero, the leakage process tends to stop, and the SCP in the B-annulus reaches a stable state. Considering that the leaked gas is grouped and leaks to the wellhead annulus for accumulation, the gas leakage rate is stable within the time step of (*i* – 1) Δ*t*∼*i*Δ*t* (*i* = 1, 2, 3···), so it can be obtained from eq 11.

12 $$Q_{\mathrm{vi}}=\frac{10^{-18}WH^{3}\left(p_{f}-p_{a(i-1)}-10^{-6}\rho_{l}gH_{p}\right)}{12L_{c}\eta }$$

where *Q*_vi_ is the gas leakage rate at the cement sheath interface within (*i* – 1) Δ*t*∼*i*Δ*t*, cm^3^/s; and *p*_a(*i*–1)_ is the SCP in B-annulus at the end of (*i* – 1) Δ*t*, MPa.

Furthermore, the amount of substance *n*_i_ of the leaked gas can be obtained from the equation of state of the leaked gas at the top surface of the cement sheath.

13 $$n_{i}=\frac{\left(p_{a(i-1)}+10^{-6}\rho_{l}gH_{p}\right)Q_{\mathrm{vi}}\cdot \Delta t}{Z_{i‐1}RT_{m}}$$

where *n*_i_ is the amount of substance of the leaked gas within (*i* – 1) Δ*t*∼*i*Δ*t*, mol; *T*_m_ is the temperature at the depth of the top surface of the cement sheath, K; Δ*t* is the time step, s.

The deviation factor *Z* of the leaked natural gas is obtained from the Peng–Robinson equation:^33^

14 $$Z=1+\beta -q\beta \left(\frac{Z-\beta }{\left[Z+\left(1+\sqrt{2}\right)\beta \right]\left[Z+\left(1-\sqrt{2}\right)\beta \right]}\right)$$

where *Z* is the deviation factor of the leaked natural gas, dimensionless; β is the correction term for the effect of pressure and temperature on molecular volume, dimensionless; and *q* is the correction term for intermolecular attraction, dimensionless.

Finally, considering the accumulation of leaked gas at the wellhead, the SCP in the B-annulus after the *i*-th group of gas reaches the wellhead can be obtained as:

15 $$p_{ai}=\frac{Q_{\mathrm{vi}}\cdot \Delta t\cdot Z_{0}T_{0}\overset{i}{\underset{x=0}{\sum }}n_{x}\left(p_{a(i-1)}+10^{-6}\rho_{l}gH_{p}\right)}{V_{0}n_{i}Z_{i}T_{m}}$$

where *V*_0_ is the volume of the B-annulus gas chamber at the wellhead, cm^3^; and *T*_0_ is the average temperature of the B-annulus at the wellhead, K. When the SCP in the B-annulus reaches a steady state, it should satisfy:

16 $$p_{ai}-p_{a(i-1)}\leq \varepsilon$$

where ε = 10^–6^ MPa.

The flowchart of the model solution is illustrated in Figure 5.

**Figure 5:**
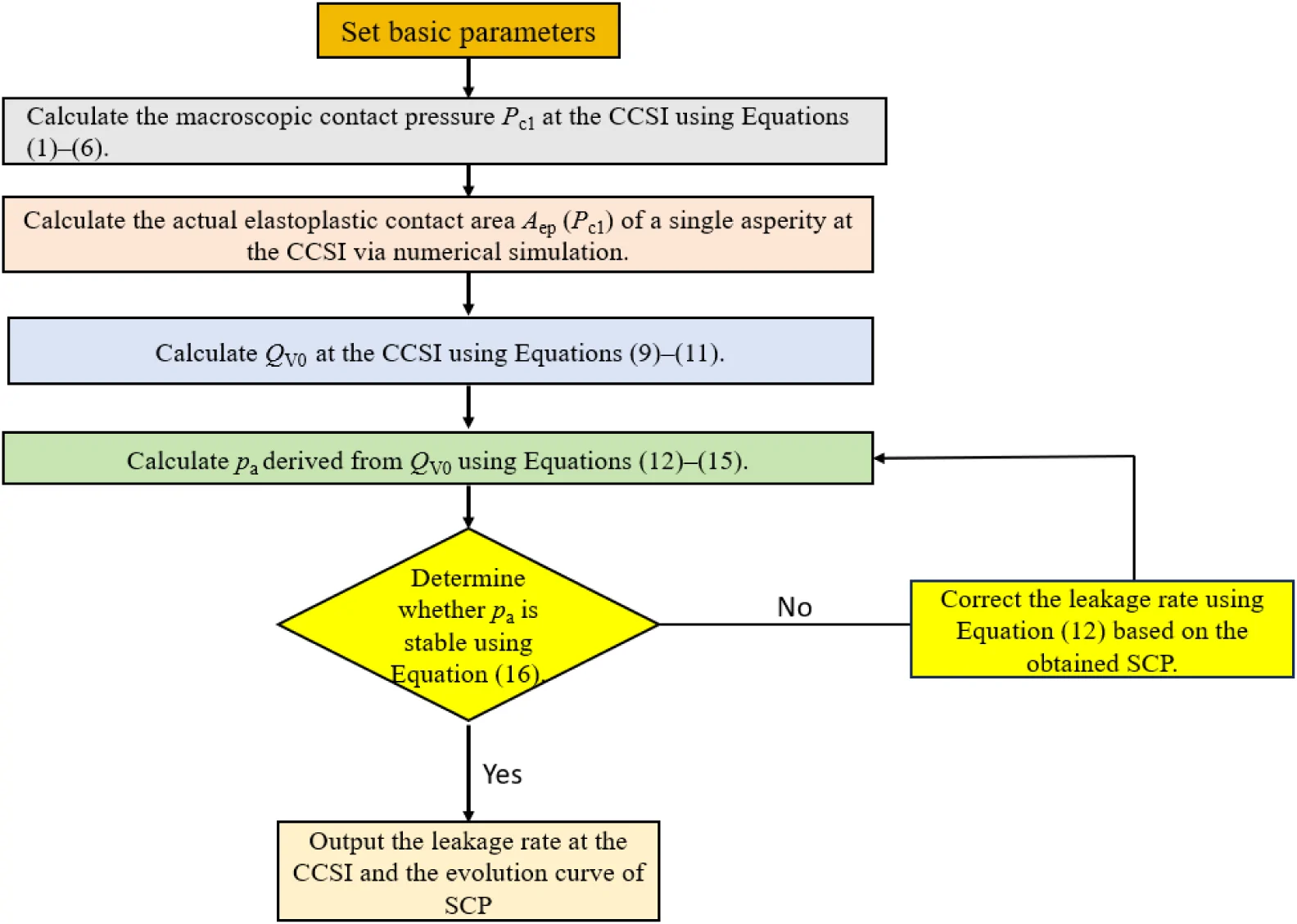
Flowchart of the model solution.

## 3 Case Analysis and Model Validation

Taking a shale gas well in western Chongqing, Sichuan Basin, as an example, case analysis and model validation are carried out. The basic parameters of the example well are listed in Table 1.

**Table 1:** Basic Parameters of a Shale Gas Well

Parameter Symbol	Parameter	Value	Unit
*a*	Inner radius of casing	57.15	mm
*b*_1_	Outer radius of casing/inner radius of cement sheath	69.85	mm
*b*_2_	Outer radius of cement sheath/inner radius of formation	107.95	mm
*d*	Outer radius of formation	1079.50	mm
*E*_s_	Elastic modulus of casing	206000	MPa
ν_s_	Poisson’s ratio of casing	0.30	dimensionless
α_s_	Thermal expansion coefficient of casing	1.22 × 10^–05^	/°C
*E*_c_	Elastic modulus of cement sheath	10000	MPa
ν_c_	Poisson’s ratio of cement sheath	0.18	dimensionless
α_c_	Thermal expansion coefficient of cement sheath	1.05 × 10^–05^	/°C
*E*_f_	Elastic modulus of formation	15000	MPa
ν_f_	Poisson’s ratio of formation	0.19	dimensionless
α_f_	Thermal expansion coefficient of formation	1.03 × 10^–05^	/°C
*p*_i_	Casing internal pressure	30	MPa
*p*_f_	Formation pressure	30	MPa
Δ*T*	Wellbore temperature variation	24	°C
σ_ts_	Tensile strength of cement sheath	3	MPa
σ_cs_	Compressive strength of cement sheath	36	MPa
*L*_c_	Axial length of cement sheath	2600	m
η	Dynamic viscosity of natural gas	1.37 × 10^–5^	Pa·s
ρ_i_	Density of annular protective fluid	1.3	g/cm^3^
*H*_p_	Liquid column height of annular protective fluid	1107	m
*R*_a_	Surface roughness of cement sheath	20	μm

First, the contact pressure *p*_c1_ at the CCSI was calculated using the model in Section 2.1. Then, the evolution of the microscopic contact state of asperities with *p*_c1_ was simulated via the method in Section 2.2, with the results shown in Figure 6.

**Figure 6:**
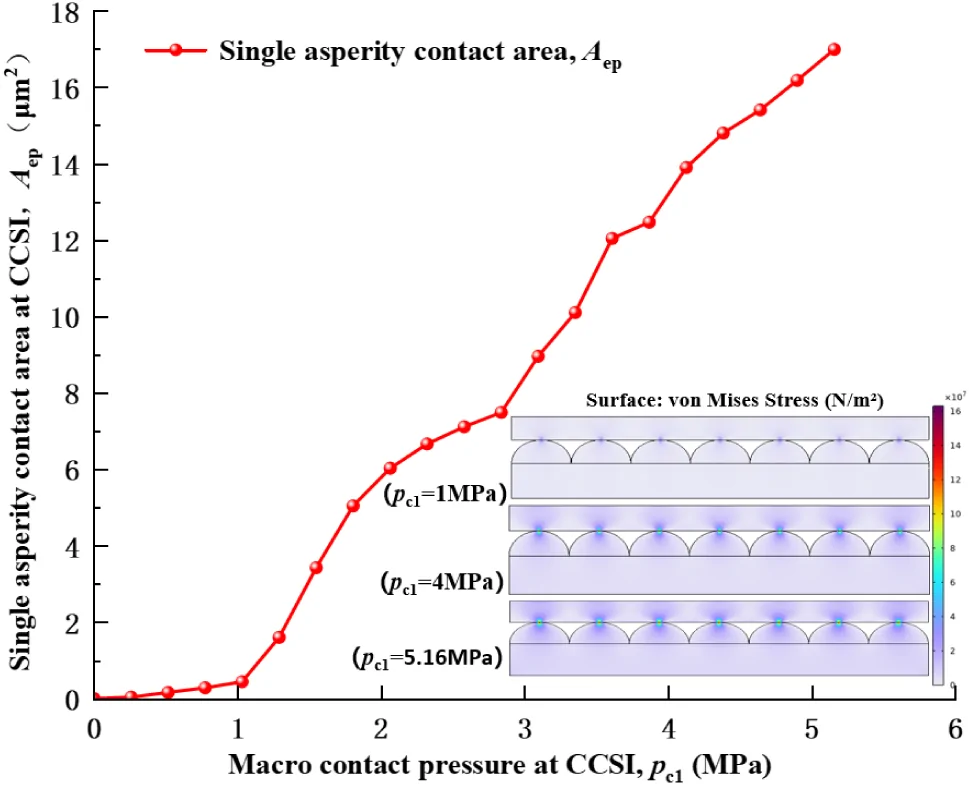
Relationship between the contact area of asperities and macro contact pressure at the CCSI.

It can be seen from Figure 6 that as *p*_c1_ increases, the contact area of asperities at the CCSI gradually increases, which directly leads to a decrease in the cross-sectional area of microscopic leakage channels at the interface. As *p*_c1_ increases from 0 to 5.16 MPa, the contact area of a single asperity rises from 0 μm^2^ to 16.992 μm^2^. The cross-sectional area of microscopic leakage channels along the circumferential direction at the CCSI decreases from 1.88 × 10^6^ μm^2^ to 1.42 × 10^6^ μm^2^, representing a reduction of 24.5%. As both the casing internal pressure and wellbore temperature decrease, the contact pressure at the CCSI is reduced, resulting in an increase in the cross-sectional area of interfacial leakage channels, a decrease in gas leakage resistance across the interface, and an increase in gas leakage rate. This is consistent with the obvious leakage bubbles observed in relevant experiments during casing pressure reduction and wellbore cooling stages.^5,6^

Figure 7 shows the calculated coupling prediction curve of the leakage rate at the CCSI versus SCP. At the initial moment (t = 0), the gas leakage pressure difference across the CCSI is the largest (15.9 MPa), and the corresponding interfacial gas leakage rate reaches a peak of 0.00139 cm^3^/s. As natural gas continuously leaks along the CCSI into the wellhead B-annulus and accumulates, the SCP in the B-annulus gradually increases. Meanwhile, the continuous increase in SCP in the B-annulus leads to a gradual reduction in the driving pressure difference for interfacial gas leakage, causing the gas leakage rate to decrease accordingly. This process continues until the driving pressure difference approaches zero, and the leakage ceases. For this well, the annular pressure stabilizes at 15.87 MPa after 5.27 days. Figure 7 also compares the field-measured evolution curve of the SCP in the B-annulus with the model-predicted one. The measured stable value of the SCP in the B-annulus is 14.82 MPa with a stabilization time of 5.17 days. The relative errors between the model prediction and field measurement are 7.09% for the stable annular pressure value and 1.9% for the stabilization time, which verifies that the model proposed in this paper has a high reliability.

**Figure 7:**
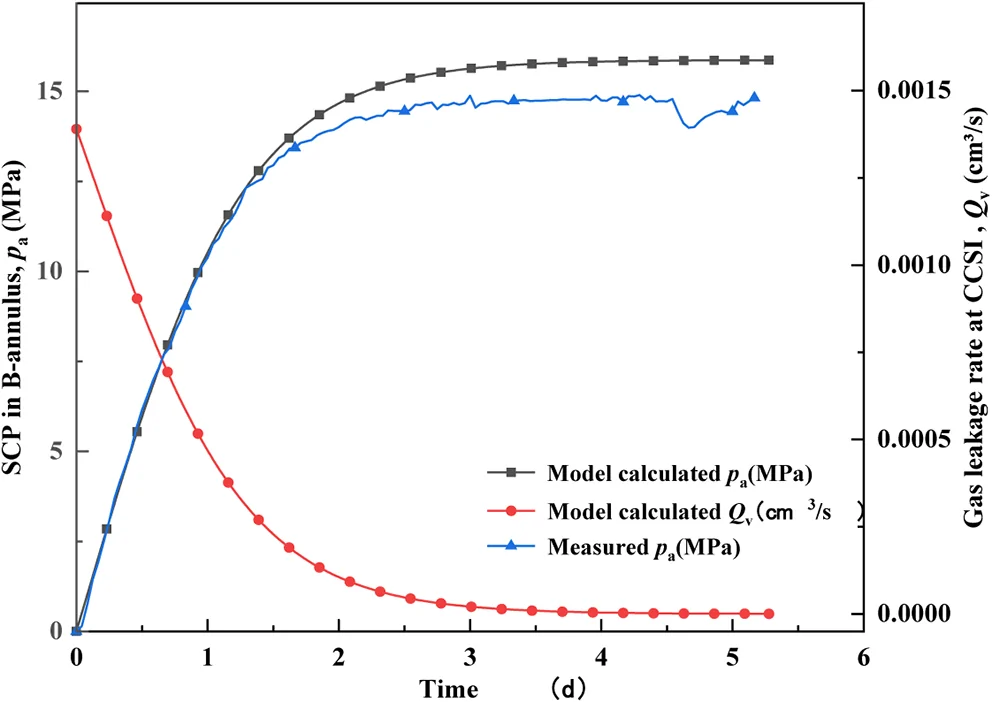
Coupling prediction curve of leakage rate at CCSI and SCP in B-annulus.

## 4 Results

A systematic parameter sensitivity analysis was conducted on the CCSI contact characteristics and SCP in the B-annulus for a shale gas well, mainly including cement-sheath material parameters, wellbore engineering parameters, and casing-cement sheath interfacial sealing parameters.

### 4.1 Cement Sheath Elastic Modulus E_c_

Figure 8(a) illustrates the effects of the *E*_c_ on the macro contact pressure at the CCSI and the contact area of a single asperity. The analysis shows that, under otherwise constant conditions, a higher *E*_c_ leads to a greater macro contact pressure at the CCSI but a smaller contact area of a single asperity. This is primarily attributed to the strong resistance to deformation of cement with a high *E*_c_. Figure 8(b) and 8(c), respectively, illustrate the evolution of gas leakage rate at the CCSI and the SCP in the B-annulus over time under different *E*_c_. The analysis shows that, influenced by the variation in the contact area of a single asperity at the CCSI, a higher *E*_c_ leads to a larger cross-sectional area of the leakage path at the interface, a higher initial gas leakage rate, a faster increase in the SCP in the B-annulus, and a shorter stabilization time. However, overall, the effect of the *E*_c_ on the evolution of the interface gas leakage rate and the SCP in the B-annulus is not significant. When the *E*_c_ increases from 4 to 16 GPa (a threefold increase), the contact area of a single asperity at the CCSI decreases from 23.18 μm^2^ to 13.19 μm^2^ (a reduction of 43.1%). As a result, the initial gas leakage rate at the CCSI increases from 1.35 × 10^–3^ cm^3^/s to 1.41 × 10^–3^ cm^3^/s, an increase of only 4%, while the stabilization time of the SCP in the B-annulus decreases from 5.44 days to 5.2 days, a reduction of only 4.1%. A cement sheath with a low *E*_c_ is beneficial for improving the stress state of the cement sheath, thereby enhancing cement sheath integrity. Additionally, it can moderately reduce the leakage rate at the CCSI and help control SCP. Therefore, a low *E*_c_ is recommended for production casing cementing in shale gas wells.

**Figure 8:**
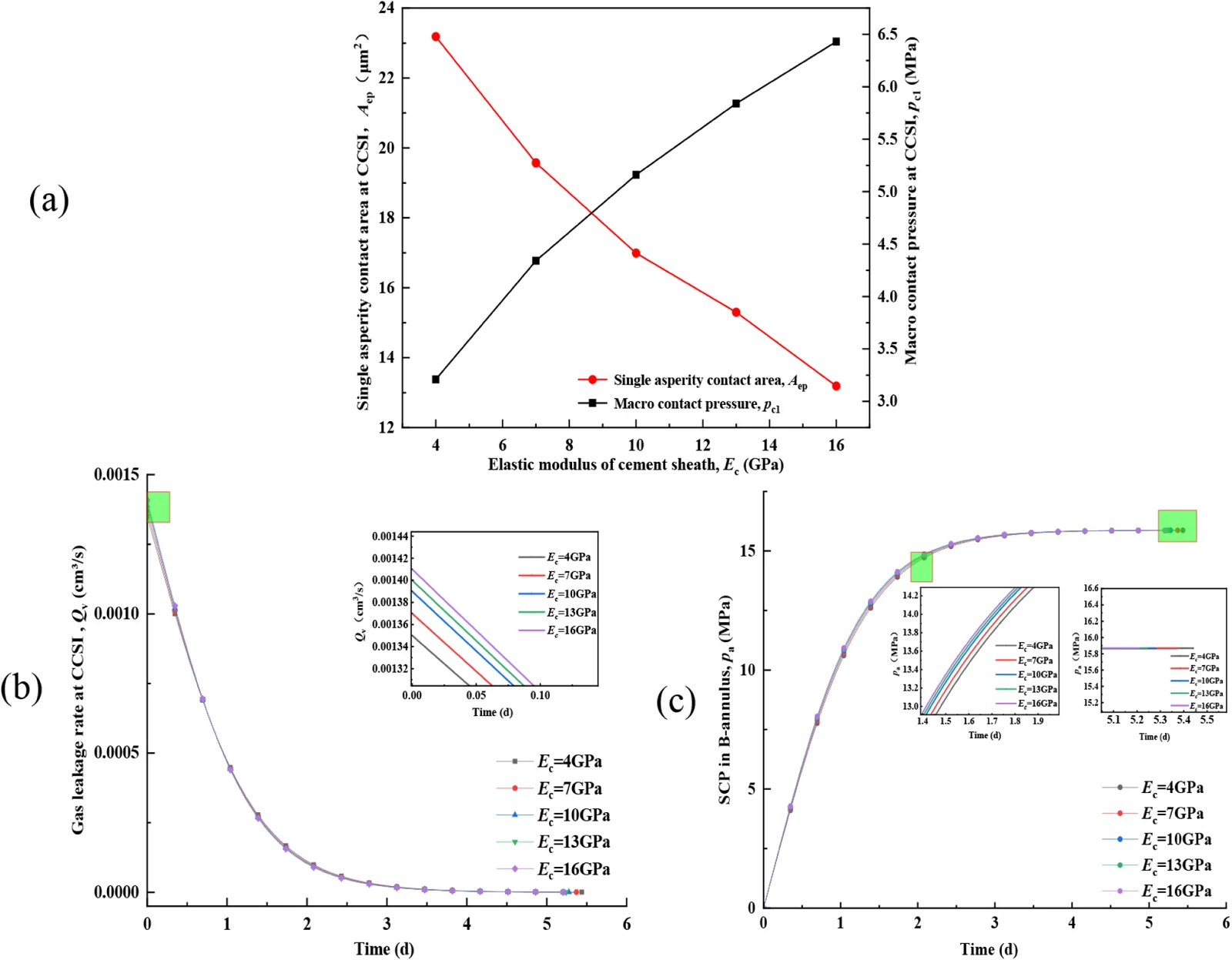
Effect of cement sheath elastic modulus *E*_c_ on CCSI contact characteristics and SCP evolution: (a) effect of *E*_c_ on the macro contact pressure and single-asperity contact area at the CCSI; (b) evolution of gas leakage rate at the CCSI with time under different *E*_c_; (c) evolution of SCP in B-annulus with time under different *E*_c_.

### 4.2 Poisson’s Ratio of the Cement Sheath *V*_c_

Figure 9(a) illustrates the effects of the *V*_c_ on the macro contact pressure at the CCSI and the contact area of a single asperity. The analysis shows that, under otherwise constant conditions, a higher *V*_c_ of the cement sheath leads to a lower macro contact pressure at the interface but a larger contact area of a single asperity. This is mainly attributed to the greater transverse deformation capacity of cement with a high *V*_c_. Figure 9(b) and 9(c), respectively, illustrate the evolution of the gas leakage rate at the CCSI and the SCP in the B-annulus over time under different *V*_c_. Overall, the *V*_c_ has no significant effect on the evolution of the gas leakage rate at the CCSI and the SCP in the B-annulus, primarily because variations in the *V*_c_ have only a minor impact on the contact area of a single asperity at the interface. When the *V*_c_ increases from 0.14 to 0.22 (an increase of 57.1%), the contact area of a single asperity at the CCSI increases only from 16.5 μm^2^ to 17.4 μm^2^ (an increase of 5.5%). Consequently, the initial gas leakage rate at the CCSI decreases from 1.395 × 10^–3^ cm^3^/s to 1.385 × 10^–3^ cm^3^/s, a reduction of only 0.7%, while the stabilization time of the SCP in the B-annulus increases from 5.27 days to 5.31 days, an increase of only 0.76%. Therefore, using a cement sheath with a high *V*_c_ can appropriately reduce the risk of micro-annulus leakage.

**Figure 9:**
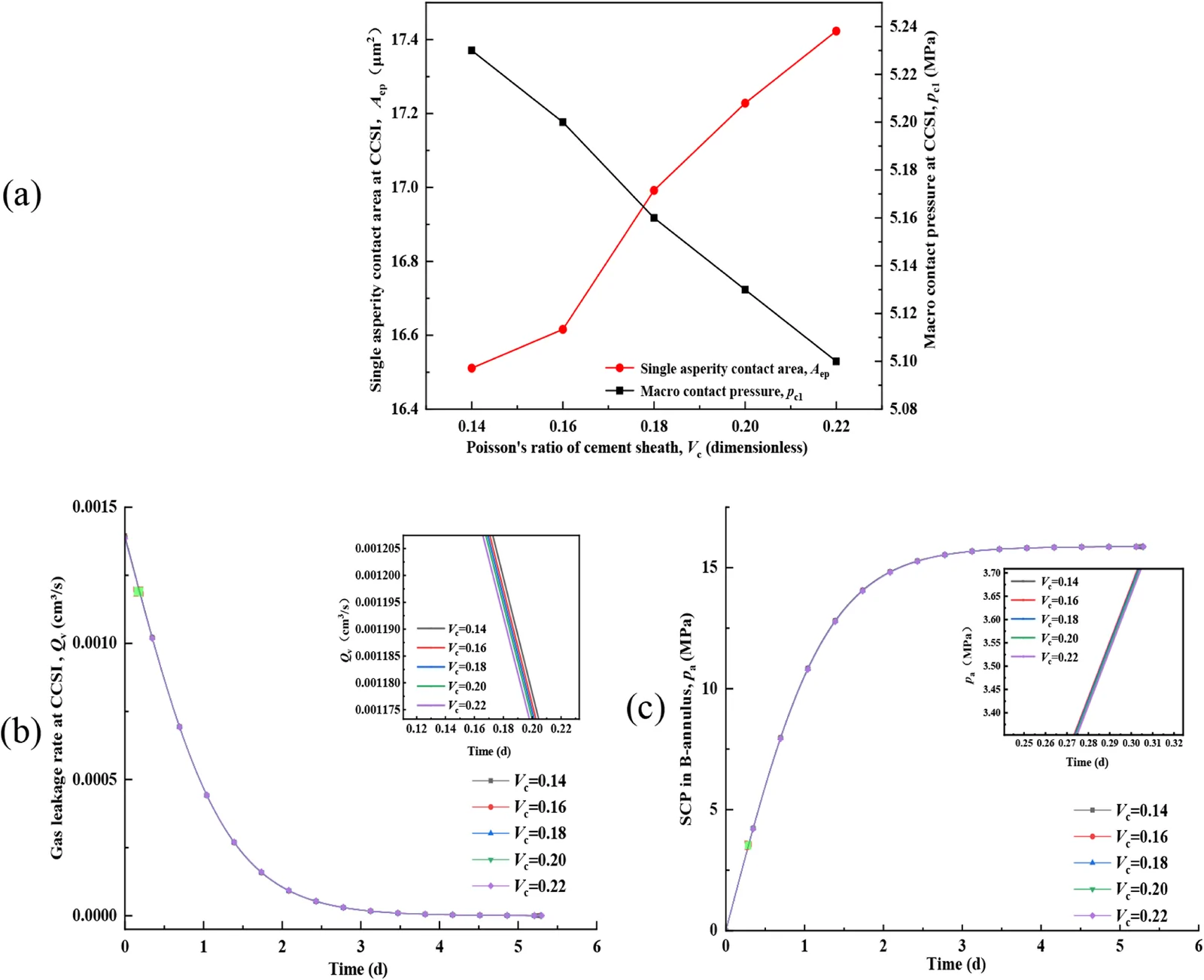
Effect of cement sheath Poisson’s ratio *V*_c_ on CCSI contact characteristics and SCP evolution: (a) effect of *V*_c_ on the macro contact pressure and single-asperity contact area at the CCSI; (b) evolution of gas leakage rate at the CCSI with time under different *V*_c_; (c) evolution of SCP in B-annulus with time under different *V*_c_.

### 4.3 Surface Roughness of the Cement Sheath *R*_a_

Figure 10(a) presents the microscopic contact simulation results at the CCSI under different *R*_a_, where the macro contact pressure at the interface is 5.16 MPa. Figure 10(b) shows the corresponding variation of the contact area of a single asperity with *R*_a_. Analysis of Figure 10(b) indicates that, under otherwise constant conditions, as *R*_a_ increases, the contact area of a single asperity at the CCSI increases in an approximately exponential manner. Correspondingly, Figure 10(a) clearly shows a significant increase in the cross-sectional area of the microscopic leakage path at the interface, which reduces the gas leakage resistance at the CCSI. Figure 10(c) and 10(d), respectively, illustrate the evolution of the gas leakage rate at the CCSI and the SCP in the B-annulus over time under different *R*_a_. The analysis shows that as *R*_a_ increases, the resulting larger microscopic leakage path at the interface implies lower gas leakage resistance, leading to a higher initial gas leakage rate at the CCSI, a faster increase in the SCP in B-annulus, and a shorter stabilization time. When *R*_a_ increases from 10 μm to 30 μm (a twofold increase), the initial gas leakage rate at the CCSI increases from 1.34 × 10^–4^ cm^3^/s to 5.04 × 10^–3^ cm^3^/s, an increase of 37.6 times, while the stabilization time of the SCP in the B-annulus decreases from 49.14 days to 2.06 days, a reduction of approximately 24 times. This indicates that *R*_a_ significantly influences the gas leakage rate at the CCSI and the evolution process of the SCP in the B-annulus. Therefore, minimizing *R*_a_ by optimizing the particle size and distribution of cement slurry additives can effectively reduce the gas leakage rate at the CCSI and mitigate the SCP in B-annulus.

**Figure 10:**
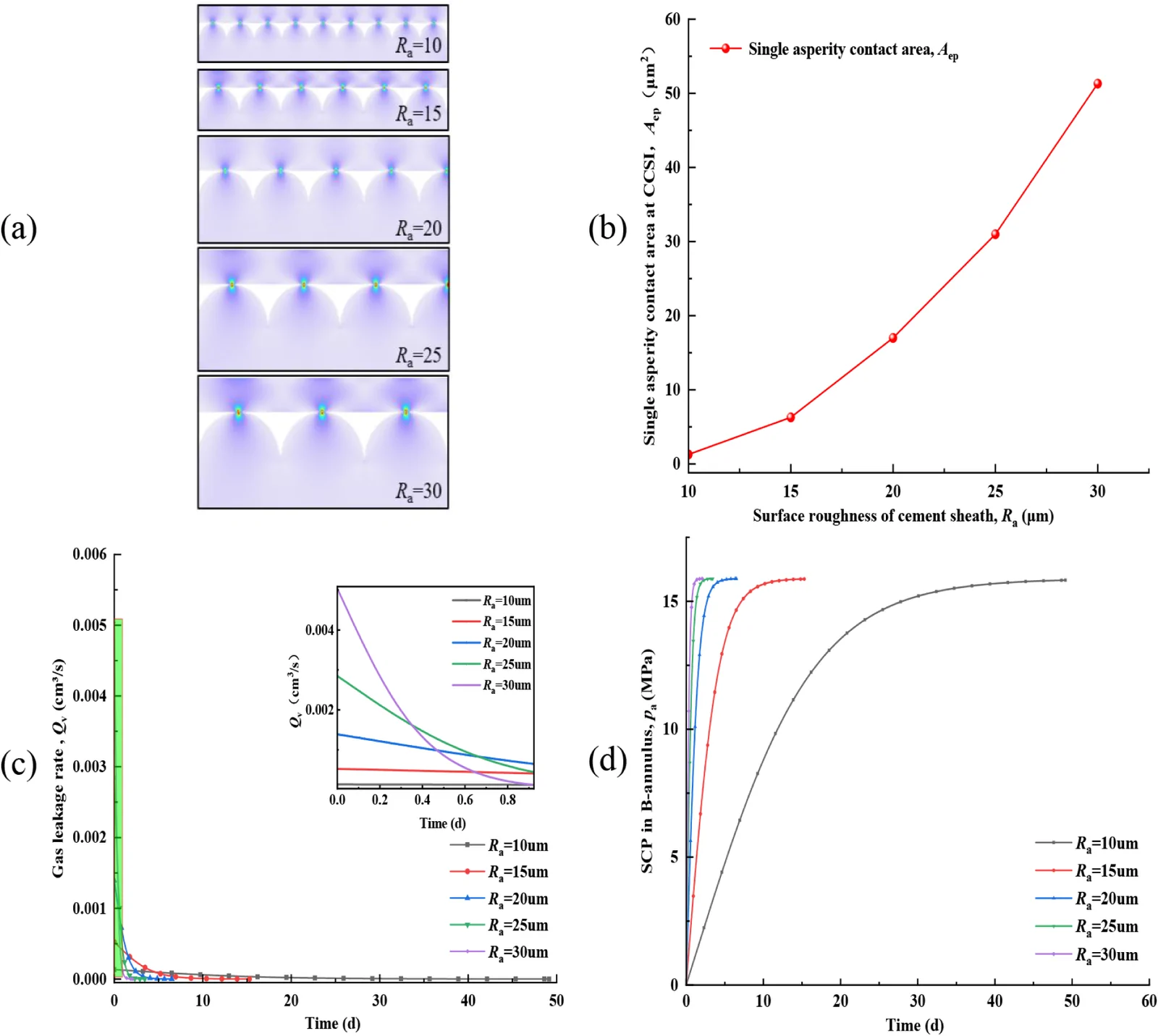
Effect of cement sheath surface roughness *R*_a_ on CCSI contact characteristics and SCP evolution: (a) microscopic contact states at the CCSI under different *R*_a_; (b) variation of single-asperity contact area with *R*_a_; (c) evolution of gas leakage rate at the CCSI with time under different *R*_a_; (d) evolution of SCP in B-annulus with time under different *R*_a_.

### 4.4 Axial Leakage Length at the CCSI *L*_c_

Figure 11(a) and 11(b), respectively, illustrate the evolution of the gas leakage rate at the CCSI and the SCP in the B-annulus over time under different *L*_c_. The analysis shows that, under otherwise constant conditions, a longer *L*_c_ results in a lower initial gas leakage rate at the interface, a slower increase in the SCP in the B-annulus, and a longer stabilization time. This is primarily because a longer *L*_c_ increases the resistance to gas flow through the leakage path at the interface, thereby reducing the gas leakage rate. When *L*_c_ increases from 1600 to 3600 m, the initial gas leakage rate through the leakage path decreases from 2.25 × 10^–3^ cm^3^/s to 1.00 × 10^–3^ cm^3^/s, a reduction of 55.6%, while the stabilization time of the SCP in the B-annulus increases from 3.45 days to 7.07 days, an increase of 105%. This indicates that variations in *L*_c_ significantly affect the gas leakage rate at the CCSI and the evolution process of the SCP in the B-annulus. Therefore, for deep shale gas wells, it is recommended that the production casing cementing operation be designed to return the cement slurry to the wellhead, thereby maximizing the length of the cemented section.

**Figure 11:**
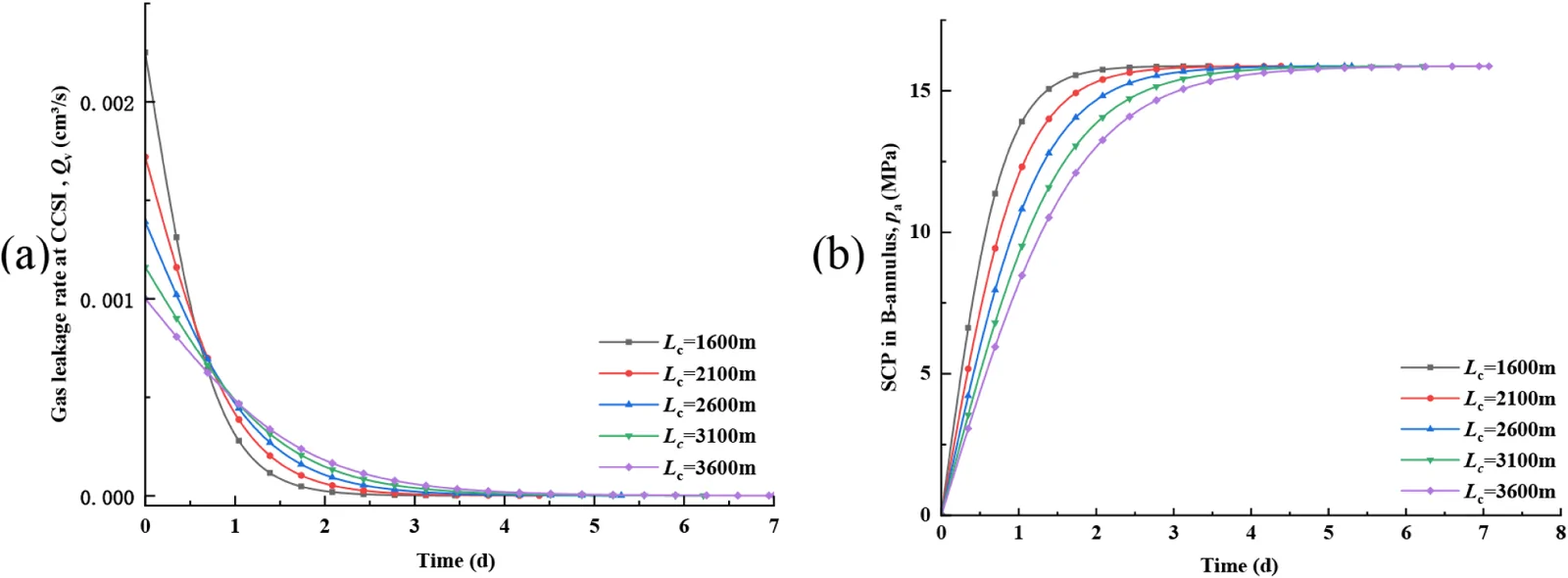
Effect of axial leakage length *L*_c_ on gas leakage and SCP evolution: (a) evolution of gas leakage rate at the CCSI with time under different *L*_c_; (b) evolution of SCP in B-annulus with time under different *L*_c_.

### 4.5 Production Casing Internal Pressure *P*_i_

Figure 12(a) illustrates the effect of *P*_i_ on the macro contact pressure at the CCSI and the contact area of a single asperity. The analysis shows that, under otherwise constant conditions, as *P*_i_ increases, the macro contact pressure at the interface increases approximately linearly, while the contact area of a single asperity increases approximately following a power law. This is primarily because an increase in *P*_i_ causes the casing-cement sheath-formation assembly to expand radially under compression, resulting in tighter contact at the interface. Figure 12(b) and 12(c), respectively, illustrate the evolution of the gas leakage rate at the CCSI and the SCP in the B-annulus over time under different *P*_i_. The analysis shows that, influenced by the variation in the contact area of a single asperity at the interface, a higher *P*_i_ leads to a lower initial gas leakage rate at the interface, a slower increase in the SCP in the B-annulus, and a longer stabilization time. When *P*_i_ increases from 10 to 50 MPa (a fourfold increase), the contact area of a single asperity at the interface increases from 4.32 μm^2^ to 47.77 μm^2^ (an increase of approximately tenfold), the initial gas leakage rate at the interface decreases from 1.5 × 10^–3^ cm^3^/s to 1.2 × 10^–3^ cm^3^/s (a reduction of 20%), and the stabilization time of the SCP in B-annulus increases from 4.94 days to 6.03 days (an increase of 22%). This indicates that variations in *P*_i_ significantly affect the evolution of the gas leakage rate at the CCSI and the SCP in the B-annulus. Although the gas leakage rate at the interface can be reduced and SCP can be controlled by increasing the production casing internal pressure *P*_i_ through regulation of gas well production rate, this often contradicts the economic extraction of shale gas wells.

**Figure 12:**
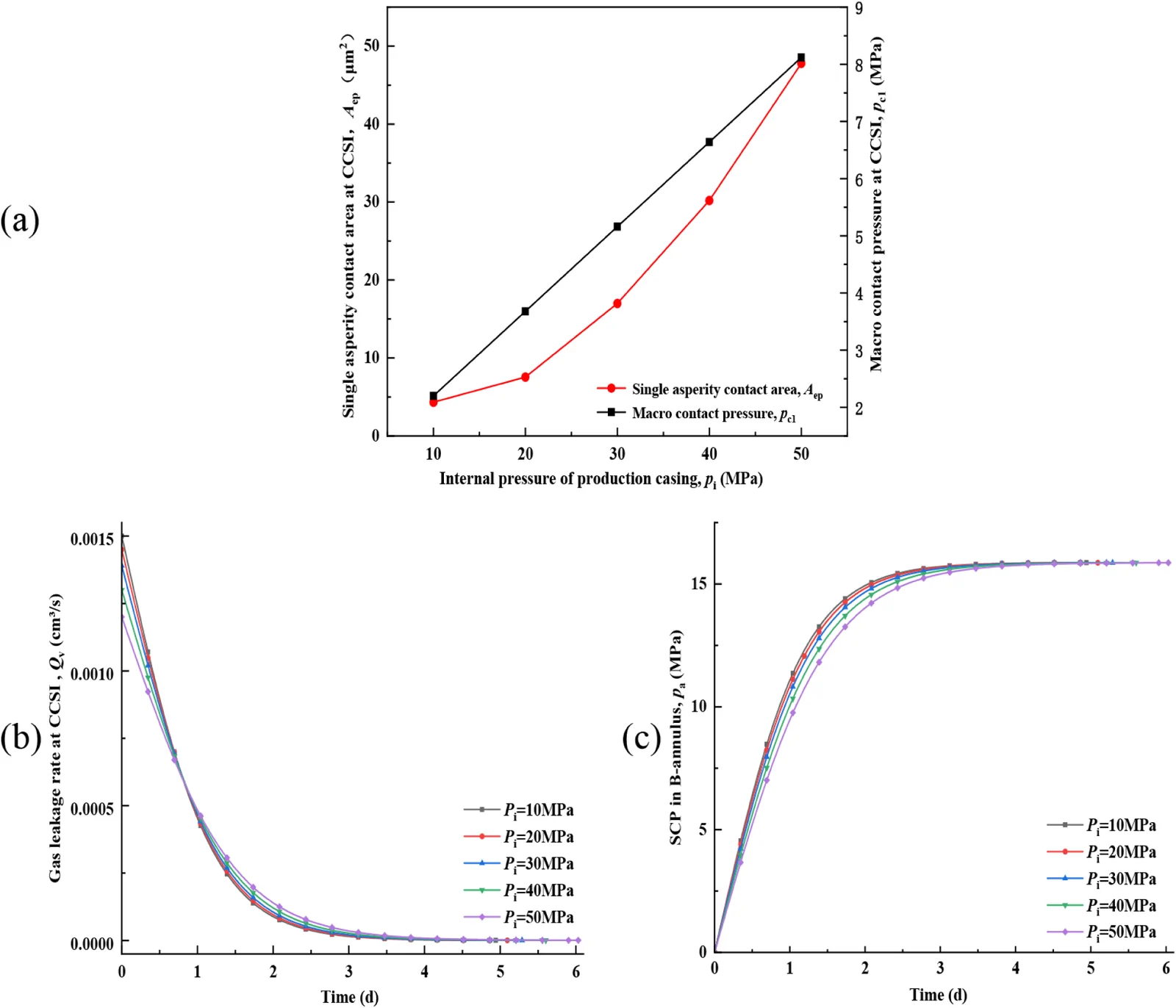
Effect of production casing internal pressure *P*_i_ on CCSI contact characteristics and SCP evolution: (a) effect of *P*_i_ on the macro contact pressure and single-asperity contact area at the CCSI; (b) evolution of gas leakage rate at the CCSI with time under different *P*_i_; (c) evolution of SCP in B-annulus with time under different *P*_i_.

### 4.6 Wellbore Temperature Change Δ*T*

Figure 13(a) illustrates the effect of Δ*T* on the macro contact pressure at the CCSI and the contact area of a single asperity. The analysis shows that, under otherwise constant conditions, as Δ*T* increases, both the macro contact pressure at the interface and the contact area of a single asperity increase approximately linearly. This is primarily because an increase in Δ*T* causes the casing-cement sheath-formation assembly to undergo radial expansion upon heating, resulting in tighter contact at the interface. Figure 13(b) and 13(c), respectively, illustrate the evolution of the gas leakage rate at the CCSI and the SCP in the B-annulus over time under different Δ*T*. The analysis shows that, influenced by the variation in the contact area of a single asperity at the interface, a greater Δ*T* leads to a lower initial gas leakage rate at the interface, a slower increase in the SCP in the B-annulus, and a longer stabilization time. When Δ*T* increases from 8 °C to 40 °C (a fourfold increase), the contact area of a single asperity at the interface increases from 11.34 μm^2^ to 26.42 μm^2^ (an increase of 1.33 times), the initial gas leakage rate through the leakage path at the interface decreases from 1.43 × 10^–3^ cm^3^/s to 1.32 × 10^–3^ cm^3^/s (a reduction of 7.7%), and the stabilization time of the SCP in B-annulus increases from 5.15 days to 5.58 days (an increase of 8.3%). Overall, Δ*T* has no significant effect on the evolution of the gas leakage rate at the casing-cement sheath interface and the SCP in the B-annulus, and to some extent, it is beneficial for reducing the interface leakage rate and controlling SCP. However, in practice, it should also be considered that an increase in wellbore temperature simultaneously raises the risk of thermally induced SCP.

**Figure 13:**
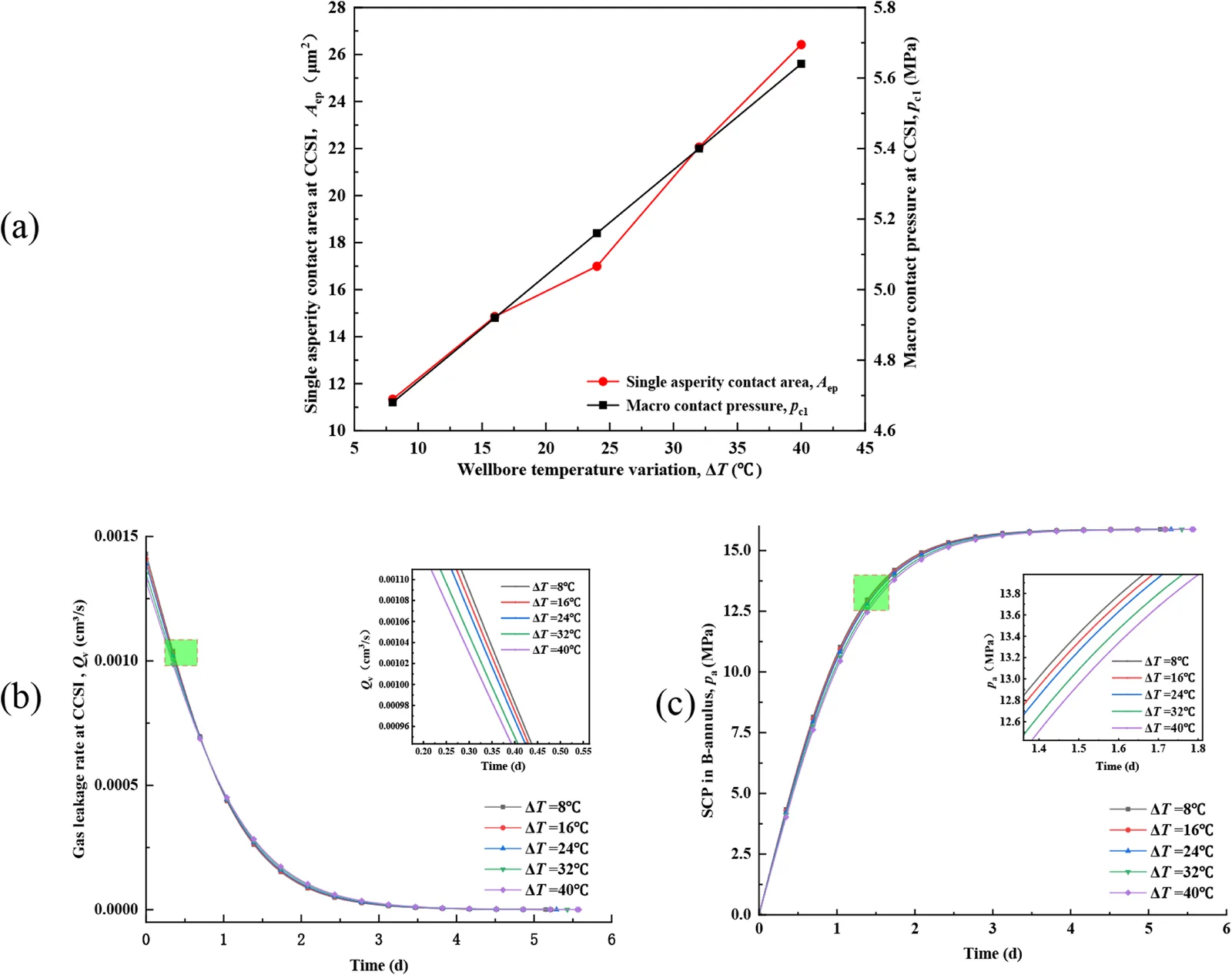
Effect of wellbore temperature change Δ*T* on CCSI contact characteristics and SCP evolution: (a) effect of Δ*T* on the macro contact pressure and single-asperity contact area at the CCSI; (b) evolution of gas leakage rate at the CCSI with time under different Δ*T*; (c) evolution of SCP in B-annulus with time under different Δ*T*.

### 4.7 Annulus Protection Fluid Density ρ_i_

Figure 14(a) and 14(b), respectively, illustrate the evolution of the gas leakage rate at the CCSI and the SCP in the B-annulus over time under different ρ_i_. The analysis shows that, under otherwise constant conditions, a higher ρ_i_ leads to a lower initial gas leakage rate at the interface, a slower increase in the SCP in the B-annulus, a lower final stabilized pressure, and a shorter stabilization time. This is primarily because a higher ρ_i_ results in a greater hydrostatic pressure of the annulus protection fluid column in the annulus, which reduces the driving pressure differential for leakage at the CCSI, while also reducing the SCP in the B-annulus required to balance the formation pressure. When ρ_i_ increases from 1100 kg/m^3^ to 1500 kg/m^3^, the initial gas leakage rate through the leakage path at the CCSI decreases from 1.49 × 10^–3^ cm^3^/s to 1.08 × 10^–3^ cm^3^/s, a reduction of 27.5%, the stabilized annulus pressure decreases from 17.05 to 12.34 MPa, a reduction of 27.6%, and the stabilization time decreases from 5.4 days to 5.0 days, a reduction of 7.4%. This indicates that variations in ρ_i_ significantly affect the gas leakage rate at the SCP in the B-annulus and the stabilized annulus pressure. Since ρ_i_ is primarily determined by the completion design, appropriately increasing ρ_i_ is beneficial for controlling the gas leakage rate at the CCSI and reducing the risk of SCP.

**Figure 14:**
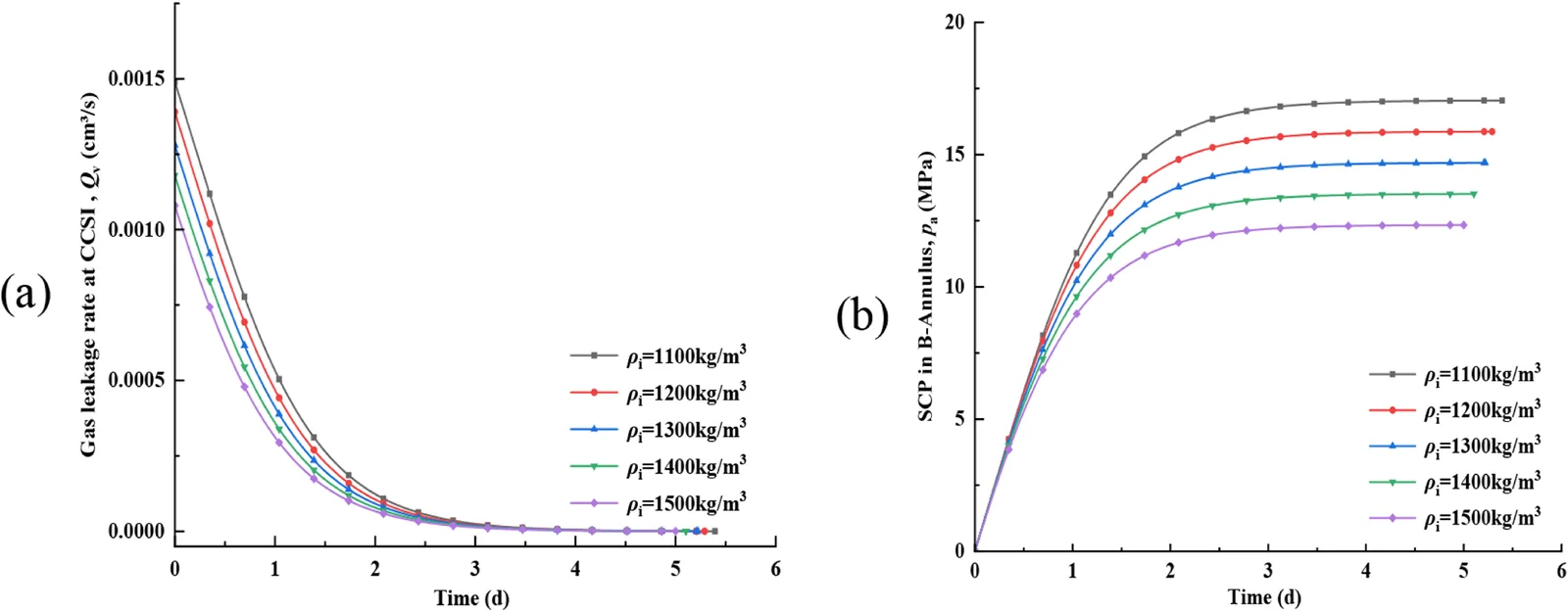
Effect of annulus protection fluid density ρ_i_ on gas leakage and SCP evolution: (a) evolution of gas leakage rate at the CCSI with time under different ρ_i_; (b) evolution of SCP in B-annulus with time under different ρ_i_.

### 4.8 Formation Pressure Change *P*_f_

Figure 15(a) and 15(b), respectively, illustrate the evolution of the gas leakage rate at the CCSI and the SCP in the B-annulus over time under different *P*_f_. The analysis shows that, under otherwise constant conditions, a lower *P*_f_ leads to a lower initial gas leakage rate at the interface, a slower increase in the SCP in B-annulus, a lower final stabilized pressure, and a shorter stabilization time. This is primarily because a lower *P*_f_ results in a smaller driving pressure differential for gas leakage at the CCSI, while also reducing the annulus pressure required to balance the formation pressure. When *P*_f_ decreases from 30 to 18 MPa, the initial gas leakage rate at the CCSI decreases from 1.39 × 10^–3^ cm^3^/s to 3.9 × 10^–4^ cm^3^/s, a reduction of 71.9%, the stabilized SCP in the B-annulus decreases from 15.9 to 3.9 MPa, a reduction of 75.5%, and the stabilization time decreases from 5.29 days to 3.79 days, a reduction of 28%. This indicates that variations in *P*_f_ significantly affect the gas leakage rate through the leakage path at the CCSI and the evolution process of annulus pressure. As shale gas wells continue to produce and formation pressure declines, the risk of SCP will gradually decrease.

**Figure 15:**
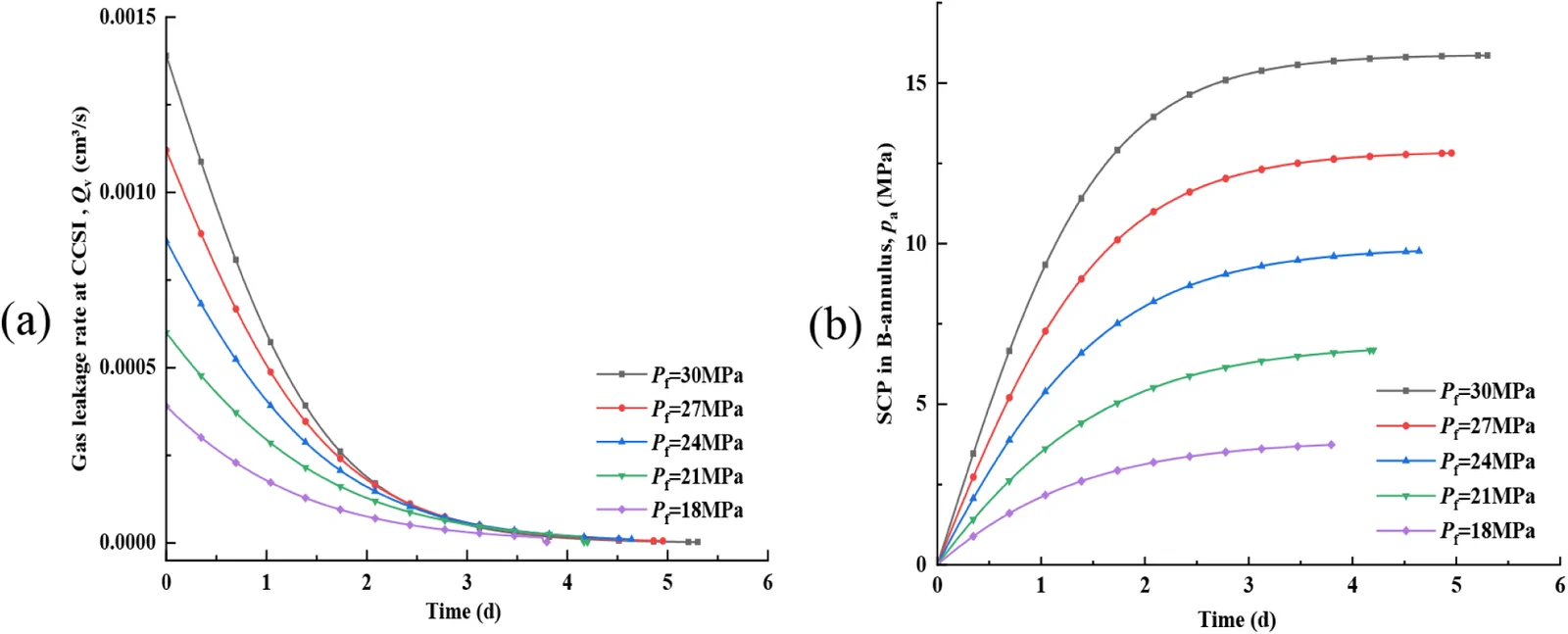
Effect of formation pressure *P*_f_ on gas leakage and SCP evolution: (a) evolution of gas leakage rate at the CCSI with time under different *P*_f_; (b) evolution of SCP in B-annulus with time under different *P*_f_.

## 5 Discussion

The B-annulus SCP prediction model developed in this study for microannular leakage at the casing-cement sheath interface elucidates the physical mechanism of SCP evolution governed by the coupled effects of the interfacial leakage rate and gas-induced pressure accumulation at the wellhead. The systematic parametric sensitivity analysis demonstrates that the mechanical properties of the cement sheath, interfacial parameters, and wellbore engineering parameters exert coupled effects on both the microannular leakage rate and the SCP evolution. Moreover, the proposed model can generate SCP evolution curves under complex input conditions, thereby enabling effective prediction of SCP induced by microannular leakage at the casing-cement sheath interface.

However, the present model considers only B-annulus SCP induced by a single leakage pathway at the casing-cement sheath interface. Under field conditions, the B-annulus SCP may result from interfacial microannular leakage, gas seepage through the cement-sheath matrix, thermal expansion, or the combined effects of multiple mechanisms. Therefore, the field application of the proposed model should involve preliminary source identification, parameter calibration, and an inverse analysis. First, the characteristic SCP evolution curves associated with the cement-matrix seepage and interfacial microannular leakage can be compared. Because these two leakage mechanisms differ in leakage path geometry and flow resistance, they are expected to exhibit different pressure buildup rates, stabilized SCP values, and times to stabilization. When combined with postfracturing cement evaluation logs, these differences can be used to assess whether interfacial microannular leakage is the dominant cause of B-annulus pressure buildup. Second, forward simulations can be performed for potential leakage from different gas-bearing intervals to generate reference SCP evolution curves at specified formation depths and pressures. By integration of these simulated curves with field-monitored SCP data and compositional analysis of the annular gas, inverse analysis can be conducted to determine whether the leaking gas originates from a shallow gas-bearing formation or the production interval. In addition, uncertain model inputs, including the annular protective fluid level, wellhead temperature, and initial B-annulus pressure, should be calibrated against field measurements whenever possible. This integrated workflow provides a practical basis for source identification, quantitative diagnosis, risk assessment, and management of SCP induced by cement sheath leakage.

The proposed model integrates elastoplastic normal microcontact analysis of the casing-cement sheath interface with fluid sealing theory to establish a quantitative linkage between the microscale interfacial leakage mechanism and the macroscopic buildup of annular pressure at the wellhead. By explicitly accounting for interfacial microannular leakage, the model extends existing cement sheath leakage-induced SCP models that primarily represent leakage through the equivalent permeability of the cement matrix. It therefore provides a useful theoretical and computational framework for the prediction, diagnosis, risk assessment, and management of annular pressure buildups in shale gas wells. Nevertheless, several limitations remain. Future work should incorporate the coupled effects of cement-matrix seepage and interfacial microannular leakage, develop higher-fidelity representations of three-dimensional interfacial microcontact behavior, and improve the treatment of downhole gas flow by accounting for real-gas compressibility and density variations along the leakage pathway.

## 6 Conclusions

(1) Based on the quantitative characterization of the microscopic leakage path at the CCSI through microscopic contact simulation, and considering the variation in gas leakage rate at the interface and the accumulation effect at the wellhead, this paper proposes a coupled prediction model for the microscopic gas leakage rate at the CCSI and the SCP in B-annulus in shale gas wells. The relative errors between the model predictions and field measurements for the stabilized SCP in B-annulus and the stabilization time are 7.09% and 1.9%, respectively, providing a reliable prediction method for revealing the physical mechanism of the SCP in B-annulus induced by leakage at the CCSI.

(2) Systematic parameter sensitivity analysis shows that cement sheath interface parameters and wellbore engineering parameters are the dominant factors affecting the gas leakage rate at the interface and the evolution of SCP, while cement sheath material parameters have no significant influence. This is primarily attributed to the combined effect of gas leakage resistance at the interface and the driving pressure differential.

1) A higher *E*_c_ and a lower *V*_c_ result in smaller contact deformation of interface asperities, leading to a larger cross-sectional area of the interface gas leakage path and lower leakage resistance. Consequently, the initial gas leakage rate at the interface increases, the SCP in the B-annulus rises more rapidly, and the stabilization time becomes shorter. However, overall, the influence of these two parameters on the evolution of the SCP is not significant.

2) Cement sheath interface parameters have a significant impact on the evolution of SCP. A larger *R*_a_ and a smaller *L*_c_ result in lower gas leakage resistance at the interface, leading to a higher initial gas leakage rate, a faster increase in the SCP in the B-annulus, and a shorter stabilization time.

3) Higher *P*_i_ and Δ*T* lead to a larger contact area of interface asperities, which in turn results in a smaller cross-sectional area of the interface gas leakage path and greater leakage resistance. Consequently, the initial gas leakage rate at the interface decreases, the SCP in the B-annulus rises more slowly, and the stabilization time becomes longer. Between these two factors, an increase in *P*_i_ has a more pronounced effect on the evolution of SCP compared to an increase in Δ*T*.

4) A higher ρ_i_ and a lower *P*_f_ result in a smaller driving pressure differential for gas leakage at the interface, leading to a lower initial gas leakage rate, a slower increase in the SCP in the B-annulus, a shorter stabilization time, and a lower final stabilized pressure, thereby reducing the risk of SCP in the gas well.

(3) To reduce the gas leakage rate at the CCSI and control the risk of the SCP in the B-annulus in shale gas wells, it is recommended that, on one hand, *R*_a_ be minimized and the effective sealed length of the cemented section be increased to enhance gas leakage resistance at the interface; on the other hand, a cement sheath with a low *E*_c_ and a high *V*_c_ be used for cementing to increase the contact deformation capacity of interface asperities, thereby reducing the cross-sectional area of the leakage path; in addition, ρ_i_ be appropriately increased to reduce the driving pressure differential for gas leakage at the interface and the stabilized annulus pressure.
